# Investigation of the Effects of MT on the Antioxidant Capacity of *Impatiens walleriana* Under Water Deficit In Vitro

**DOI:** 10.3390/plants15142129

**Published:** 2026-07-10

**Authors:** Snežana Milošević, Dragana Antonić Reljin, Marija J. Milovančević, Aleksandar Cingel, Milana Trifunović-Momčilov, Marija P. Marković, Angelina Subotić

**Affiliations:** Department of Plant Physiology, Institute for Biological Research “Siniša Stanković”–National Institute of the Republic of Serbia, University of Belgrade, Bulevar Despota Stefana 142, 11108 Belgrade, Serbia; dragana.antonic@ibiss.bg.ac.rs (D.A.R.); marija.djuric@ibiss.bg.ac.rs (M.J.M.); cingel@ibiss.bg.ac.rs (A.C.); milanag@ibiss.bg.ac.rs (M.T.-M.); marija.nikolic@ibiss.bg.ac.rs (M.P.M.); heroina@ibiss.bg.ac.rs (A.S.)

**Keywords:** enzymes activity, *Impatiens walleriana*, melatonin, oxidative stress indicators, water deficit

## Abstract

*Impatiens walleriana* is an ornamental plant highly susceptible to water deficit. Given the multifunctional role of melatonin (MT) in plant protection, this study evaluated the efficacy of MT treatment on plant during water deficit in alleviating stress. Shoots were micropropagated in vitro on media supplemented with MT (0, 10, 50, 100 and 150 µM), alone or combined with 3% polyethylene glycol (PEG). Morphological, physiological, and biochemical parameters were analysed after treatment. Treatment of shoots with 10 µM MT under optimal conditions resulted in a slower root growth and development, increased DPPH content and maximum superoxide dismutase activity. Shoots treated with a higher concentration of MT showed reduced values for all morphological parameters, while 150 µM MT had an inhibitory effect on scarification and induced the highest values for H_2_O_2_, total phenolic, and DPPH contents, as well as catalase and peroxidase activity. Water deficit alone significantly reduced shoot height, shoot and root fresh weight, photosynthetic pigment content, and DPPH activity, while it increased osmoprotectants, H_2_O_2_, and peroxidase activity. Plants treated with 50 μM MT during water deficit almost did not develop roots, and the application of higher concentrations of MT (100 and 150 μM) reduced shoot development with increased proline content, reduced POX activity, and inhibited root formation. This is the first study to investigate the effects of MT on the antioxidant capacity of *I. walleriana* under water deficit and provides guidance for future research on stress-tolerance mechanisms.

## 1. Introduction

Environmental variations resulting from global climate change significantly affect the future of agriculture. One of the common climate extremes globally is drought, which is also among the most serious challenges facing humanity today [1].

Drought conditions frequently lead to water deficit in plants, resulting in a wide range of morphological, physiological, biochemical, cellular, and molecular alterations, ultimately causing significant yield losses ranging from 30 to 90%, depending on the plant species, developmental stage, and duration of stress [2,3]. In the early stages of plant development, drought stress adversely affects seed germination, stem and root elongation, and overall development [4]. Drought causes tissue dehydration and inhibits cell division, elongation, and differentiation, thereby slowing basic physiological and metabolic processes [5].

A common byproduct of aerobic metabolism in plants and other organisms is reactive oxygen species (ROS), which act as signalling molecules activating defence mechanisms [6]. ROS production has been confirmed in cell membranes, the apoplast, and organelles with high oxidative activity [7,8]. There are four main forms of ROS; hydrogen peroxide (H_2_O_2_), singlet oxygen (O_2_^1^), superoxide radicals (O_2_^•−^), hydroxyl radicals (OH•) [7,9]. Under optimal conditions, ROS are maintained at relatively low levels due to a balance between their production and removal. When ROS production exceeds the capacity of the antioxidant system, homeostasis is disrupted, leading to their accumulation and oxidative stress, a common consequence of plant exposure to stress conditions. The consequences of oxidative stress are photoinhibition, structural damage to proteins and DNA through oxidative modifications, enzyme inactivation, lipid peroxidation, reduced cell division rate, and ultimately reduced plant growth [3,10].

To enhance drought tolerance in plants, many exogenously applied substances have demonstrated positive effects, including melatonin (N-acetyl-5-methoxytryptamine). Melatonin was discovered in the bovine pineal gland in 1958 [11]. As an important chronobiological hormone, it influences processes such as circadian rhythms, sleep, and the endocrine and immune systems in mammals. In plants, melatonin (MT) was first reported in Japanese morning glory (*Pharbitis nil*) and the fruits of *Solanum lycopersicum* more than thirty years ago [12,13]. Subsequently, MT was found in seeds, roots, shoots, leaves, bulbs, flowers and fruits [14,15,16,17]. Melatonin has been classified as a novel plant hormone [18]. It has a low molecular weight and an amphiphilic or amphipathic character, and therefore can easily pass through the cell membrane and distribute into the cytosol and nucleus [19]. In plants, melatonin is synthesised within chloroplasts and mitochondria [20,21]. The subcellular localization of the proteins involved in MT synthesis varies significantly among different plant species and growth stages, suggesting the existence of multiple synthetic pathways [19,22]. Numerous studies have demonstrated the various physiological functions of MT in plants [23,24,25,26]. Melatonin influences seed germination and rooting, photosynthetic efficiency, plant sugar metabolism, the redox network, antioxidant defence systems, primary and secondary metabolism, regulates the internal biological clock (including flowering, fruit ripening, and senescence), interacts with phytohormones, affects gravitropism, and acts as an endogenous biostimulant against abiotic or biotic stressors. In plants exposed to stress, it increases yield and delays apoptosis [22,27,28,29]. The key function of MT is to regulate physiological processes during plant growth and development under drought stress [16,30,31,32,33]. Melatonin regulates plant stress responses directly, by inhibiting the accumulation of ROS and reactive nitrogen species (RNS), and indirectly, by affecting stress response pathways, and activates the gene expression of stress response and antioxidative systems [20,29,31]. In addition, MT application reduces oxidative damage caused by drought in many plants, such as *Zea mays* [34], *Coffea arabica* [35], *Glycine max* [36], two species of *Salvia* [37], *Lippia citriodora* [38], *Solanum lycopersicum* [39], and *Morinda citrifolia* [40]. Treatment with 100 and/or 200 μM MT significantly helped several plant species overcome the negative effects of drought on physiological and biochemical traits [36,38,41,42]. Other studies have reported the impact of exogenous application of various concentrations of MT on plant drought tolerance in vitro [40,43].

*Impatiens walleriana* belongs to one of the largest genera *Impatiens* (Balsaminaceae), which includes more than 1000 species. Within the genus *Impatiens*, *I. hawkerii*, *I. balsamina*, and *I. campanulata* are cultivated as ornamental species, with the most common being *I. walleriana*, which has been among the most popular horticultural species worldwide since the 19th century [44]. Due to its ornamental qualities, attractive colours, and a long flowering period, *I. walleriana* is frequently used in public areas and gardens worldwide. Due to its decorative properties and relatively easy cultivation, *I. walleriana* is highly valued, and it is necessary to maintain the beauty of these plants in landscaping over the long term [45]. A lack of water in the substrate, which most often occurs during the transfer of *I. walleriana* from the producer to the market, leads to rapid tissue dehydration. The plants wilt and lose quality due to leaf and flower drop, as well as the cessation of new bud formation and development. This represents a significant problem in commercial production.

To our knowledge, no study has investigated the effect of MT on improving water-deficit tolerance in *I. walleriana*. The in vitro studies allow the detailed examination of plant responses to exogenously applied substances in a strictly controlled environment, with minimal external changes. Therefore, the present study was conducted to examine the changes in *I. walleriana* at the morphological, physiological and biochemical level after in vitro micropropagation on nutrient media containing different MT concentrations under optimal and water-deficit conditions induced by 3% PEG. Specifically, the objectives were: (i) to assess the effects of these treatments on the shoot and root growth *of I. walleriana* plants; (ii) to determine how the treatments affect the content of photosynthetic pigments; (iii) to assess the efficacy of the treatments on the content of oxidative stress indicators—proline, amino acids, H_2_O_2_, MDA (malondialdehyde), total phenolics, and DPPH activity; (iv) to determine whether the treatment alters the activity of antioxidant enzymes.

## 2. Results

### 2.1. Effects of Exogenous MT on Growth

**Figure 1:**
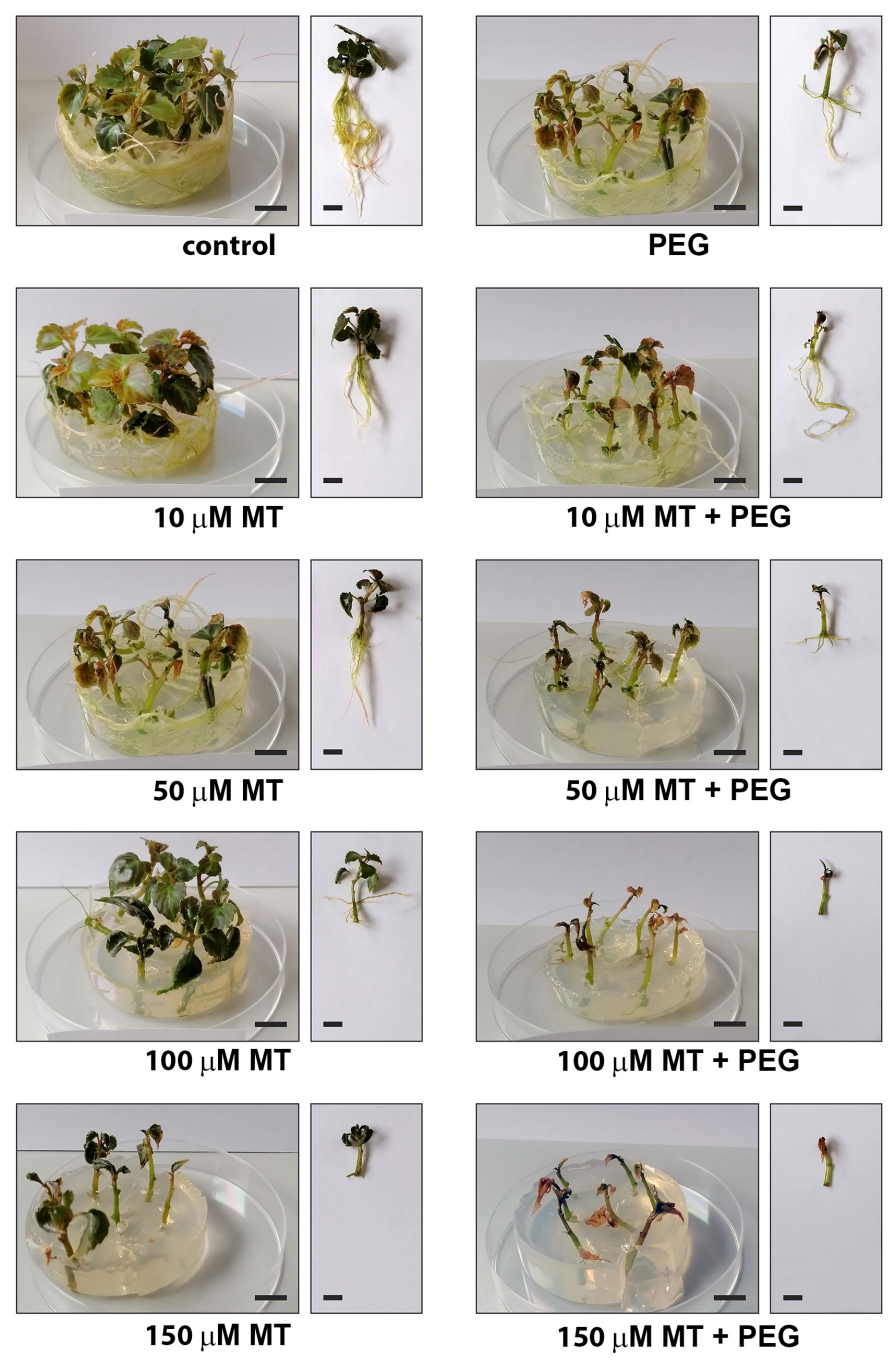
Effect of exogenously applied MT (0, 10, 50, 100 and 150 µM) on *I. walleriana* shoots grown under optimal and water-deficit (3% PEG) conditions in vitro. Bar = 1 cm.

**Figure 2:**
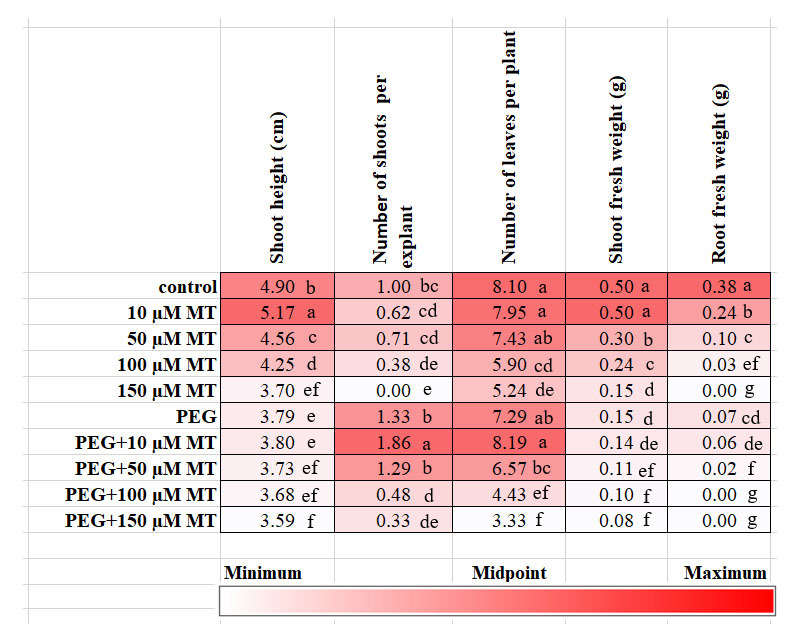
Heat map showing the effect of exogenously applied MT under optimal and water-deficit (3% PEG) conditions in vitro on growth parameters of *I. walleriana*: shoot height, number of shoots per explant, number of leaves per plant, shoot fresh weight, and root fresh weight. Data represent the mean ± SE (*n* = 21). Different letters indicate significant differences between treatments (LSD test, *p* ≤ 0.05).

Treatment with different concentrations of MT (0, 10, 50, 100 and 150 µM) and PEG-induced water deficit affected the overall growth and development of *I. walleriana* plants (Figure 1 and Figure 2).

The control plants had well-developed shoots with large, dark green leaves and formed a branched root system (Figure 1). The new shoots formed at the base of the initial stem. Treatment with 10 μM MT under optimal conditions reduced the number of newly formed shoots per explant and root growth; however, it slightly increased the height of shoots (Figure 1 and Figure 2). Similarly, plants treated with higher concentrations of MT showed decreased shoot height and root development, and induced fewer and smaller leaves (Figure 1 and Figure 2). The application of 150 µM MT significantly reduced the height and weight of fresh shoots, reduced the number of leaves and completely inhibited the formation of roots and new shoots (Figure 1 and Figure 2).

Water deficit altered the habit of *I. walleriana* plants (Figure 1 and Figure 2), led to slower shoot growth, induced fewer pale green leaves, and reduced the fresh weight of shoots and roots. Treatment of *I. walleriana* with 10 and 50 μM MT during water deficit did not alleviate PEG stress; the leaves were smaller and curled (Figure 1). The height and fresh shoot weight was within the range measured for PEG-treated shoots and significantly lower than the control (Figure 2). An increased number of newly formed shoots was observed only in shoots grown on 10 μM MT, but root development was significantly slowed. Plants treated with 50 μM MT developed almost no roots, which was also reflected in the extremely low fresh root weight values (Figure 1 and Figure 2). The combination of higher MT concentrations (100 and 150 µM) and water deficit had a negative effect on new shoot formation. The reduction in developed leaves showed a correlation with applied MT concentration. With increasing concentrations of MT (100 and 150 µM) applied during water deficit, *I. walleriana* shoots appeared progressively worse. They formed only a few rolled necrotic leaves, shoot height and weight were the lowest, and root development was completely inhibited.

### 2.2. Effect of MT Application on Photosynthetic Pigments Content Under Water Deficit

**Figure 3:**
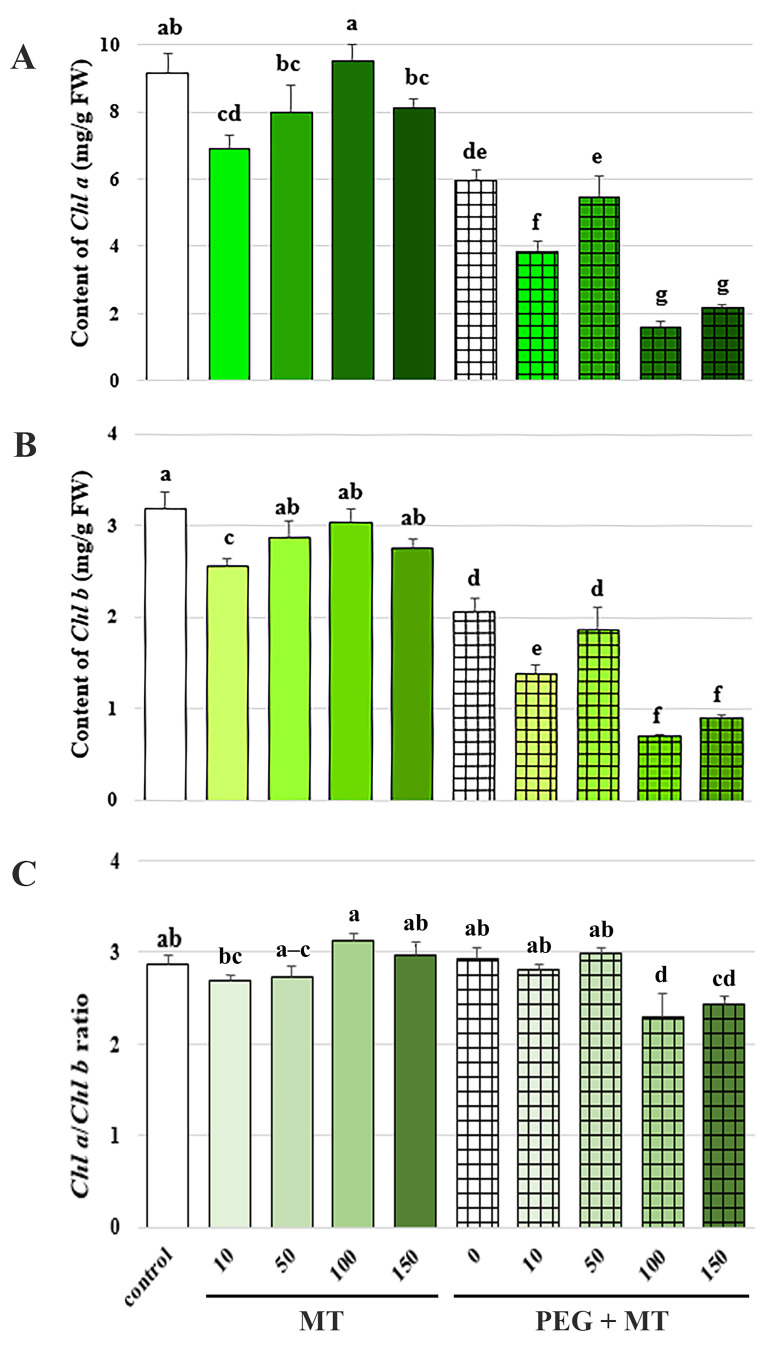
Effects of exogenously applied MT under optimal and water-deficit (3% PEG) conditions in vitro on the content of chlorophyll a *Chl a*; (**A**), chlorophyll b *Chl b* (**B**) and *Chl a*/*Chl b* ratio (**C**) in *I. walleriana* leaves. Data represent mean ± SE (*n* = 21). Different letters indicate significant differences between treatments (LSD test, *p* ≤ 0.05).

**Figure 4:**
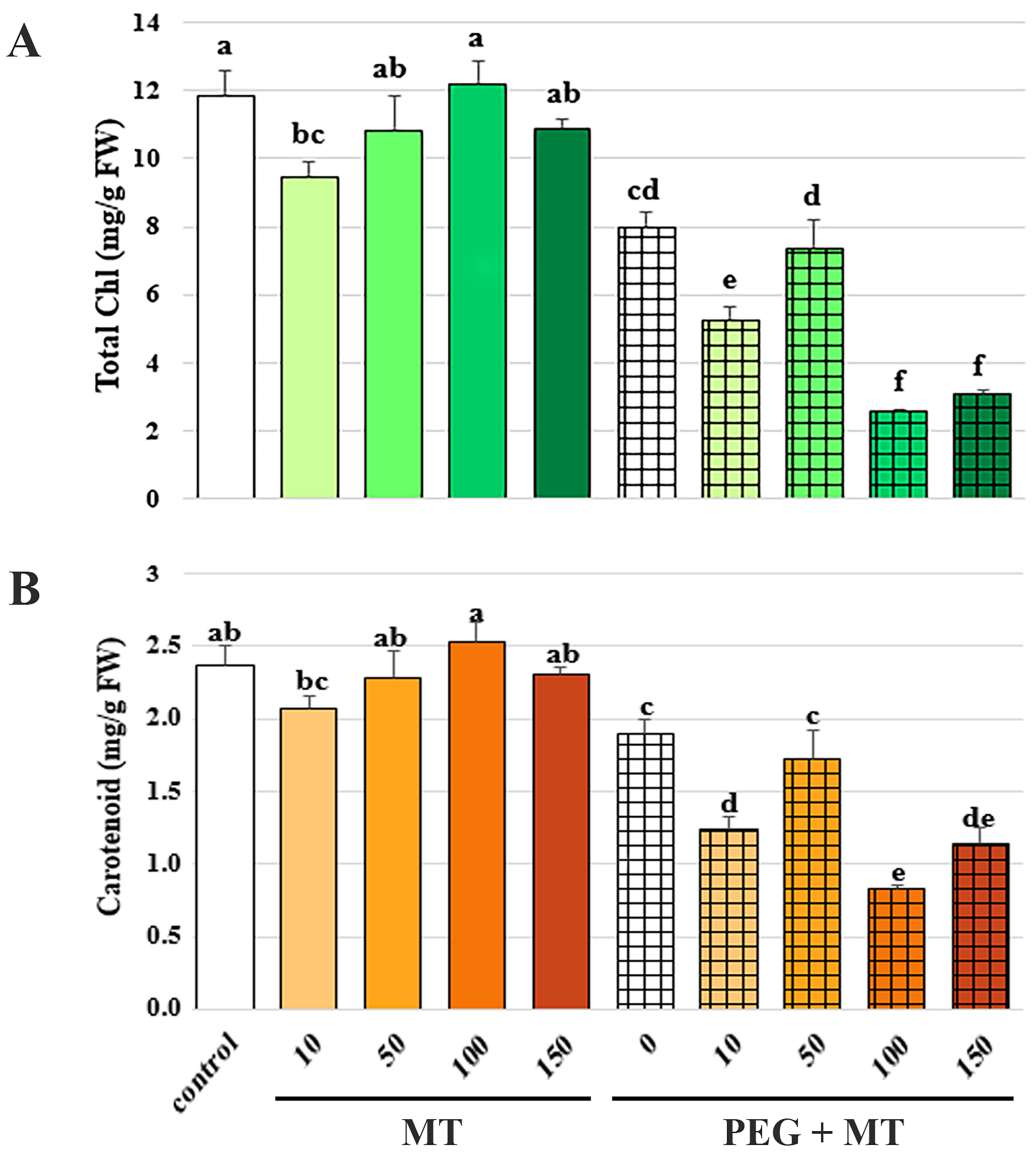
Effects of exogenously applied MT under optimal and water-deficit (3% PEG) conditions in vitro on total chlorophyll (**A**) and carotenoid content (**B**) in *I. walleriana* leaves. Data represent mean ± SE (*n* = 21). Different letters indicate significant differences between treatments (LSD test, *p* ≤ 0.05).

Treatment with 10 µM MT under optimal conditions and with osmotic stress induced by PEG alone decreased *Chl a*, *Chl b* and total chlorophyll content in *I. walleriana* leaves compared to the control (Figure 3A,B and Figure 4A). The application of 10 µM MT under water-deficit conditions resulted in even more pronounced reductions in photosynthetic pigments. The lowest values of their content were measured in *I. walleriana* leaves grown on nutrient media with 100 and 150 µM MT under water-deficit conditions.

The *Chl a*/*Chl b* ratios in the leaves of the control group and most treatments were similar (Figure 3C). A significant reduction in the *Chl a*/*Chl b* ratio was observed in *I. walleriana* after treatment with higher concentrations of MT (100 and 150 µM) under water-deficit conditions. The *Chl a*/*Chl b* ratios of the control and most treatments were similar (Figure 3C). A significant decrease in the *Chl a*/*Chl b* ratio was observed in *I. walleriana* after treatment with higher concentrations of MT (100 and 150 µM) under water-deficit conditions.

In our findings, no applied MT concentration significantly changed the carotenoid content in *I. walleriana* grown under optimal conditions compared to the control group (Figure 4B). Water deficit caused a decrease in carotenoid content, and the application of MT (especially 10, 100 and 150 µM MT) during water deficit led to a further significant decrease in carotenoid content.

### 2.3. Effects of MT Application on Proline and Amino Acids Content Under Water Deficit

**Figure 5:**
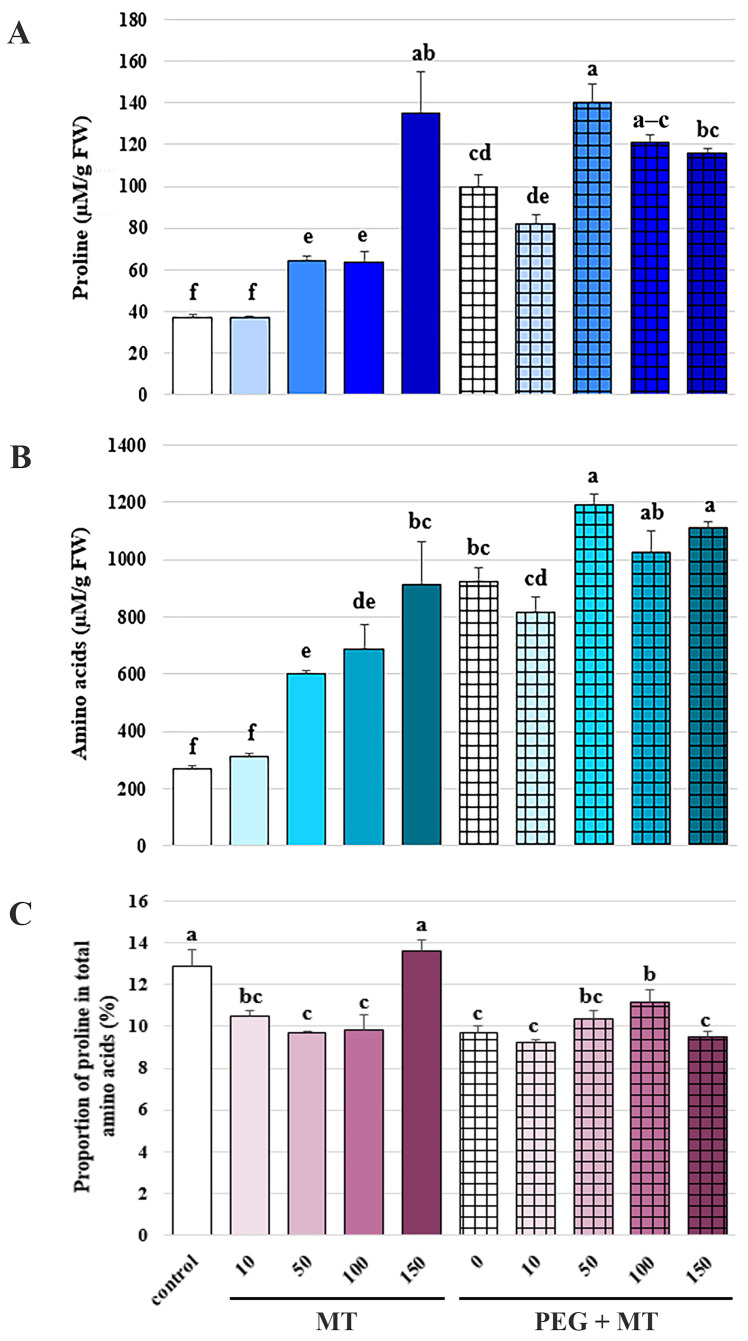
Effects of exogenously applied MT under optimal and water-deficit (3% PEG) conditions in vitro on proline (**A**), total amino acids (**B**) and the proportion of proline in total amino acids content (**C**) in *I. walleriana* shoots. Data represent mean ± SE (*n* = 21). Different letters indicate significant differences between treatments (LSD test, *p* ≤ 0.05).

To assess the extent of stress, the proline content in *I. walleriana* shoots cultivated under optimal and water-deficit conditions on media with different MT concentrations was analysed. The lowest applied MT concentration (10 µM) did not affect proline content in comparison to control (Figure 5A). The higher MT concentrations (50, 100 and 150 µM) resulted in increased proline content compared to the control plants. In *I. walleriana* shoots grown under water deficit alone and under water deficit with 10 µM MT, increased proline content was observed in comparison to control. The application of 50, 100, and 150 µM MT during water deficit resulted in increased accumulation of the osmoprotectant proline, indicating that treatment of *I. walleriana* shoots with high concentrations of MT enhances water-deficit stress.

The same trend was observed for total amino acids content (Figure 5B). The concentration of 10 µM MT did not change, while all other MT concentrations significantly elevate total amino acids content. Water deficit increased total amino acids similarly to the sole application of 150 µM MT when compared to the control. In general, all applied MT concentrations, when combined with PEG, led to a significant increase in total amino acids content.

The proportion of proline in total amino acids content was significantly lower in shoots grown on media containing 10–100 µM MT than in the control (Figure 5C). Water deficit, alone or in combination with all used MT concentrations, led to a significant reduction in the proline proportion in total amino acids content compared to the control.

### 2.4. H_2_O_2_ and MDA Content

**Figure 6:**
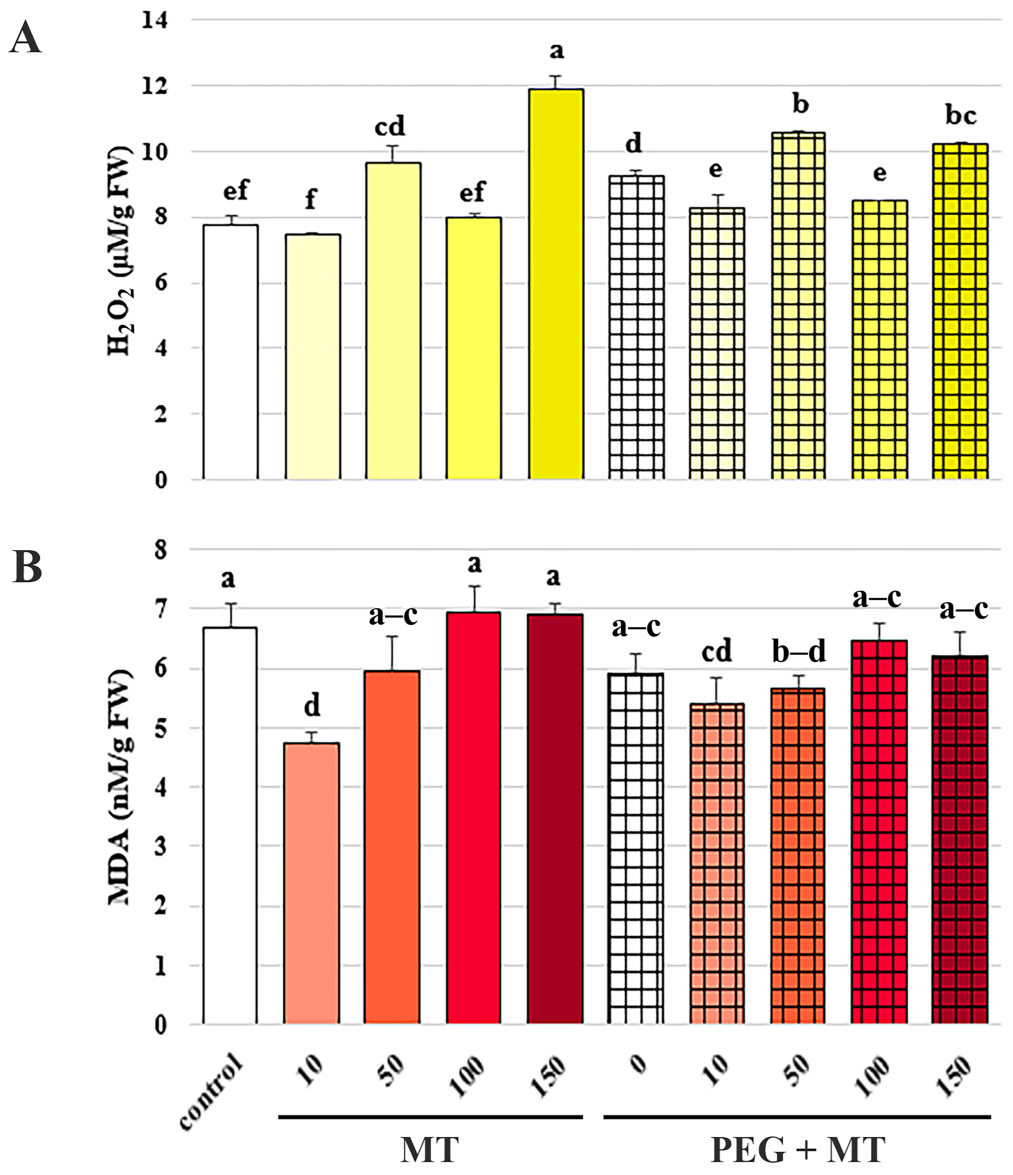
Effects of exogenously applied melatonin under optimal and water-deficit (3% PEG) conditions in vitro on H_2_O_2_ (**A**) and MDA content (**B**) in *I. walleriana* shoots. Data represent mean ± SE (*n* = 21). Different letters indicate significant differences between treatments (LSD test, *p* ≤ 0.05).

The treatments with 50 and 150 µM MT, as well as water deficit, caused a significant increase in H_2_O_2_ content compared to control *I. walleriana* shoots (Figure 6A). The H_2_O_2_ content under PEG-induced stress and MT varied depending on the applied MT concentration. The treatments with PEG and 10 or 100 µM MT did not change H_2_O_2_ content, while the concentrations of 50 and 150 µM MT significantly increased H_2_O_2_ content in comparison to control.

During the entire experimental period, MDA content did not vary a lot (Figure 6B). The only exception of a significant decrease in MDA content was observed in *I. walleriana* shoots grown on media with 10 µM MT and on media with PEG and 10 or 50 µM MT.

### 2.5. DPPH Activity and Total Polyphenol Content

**Figure 7:**
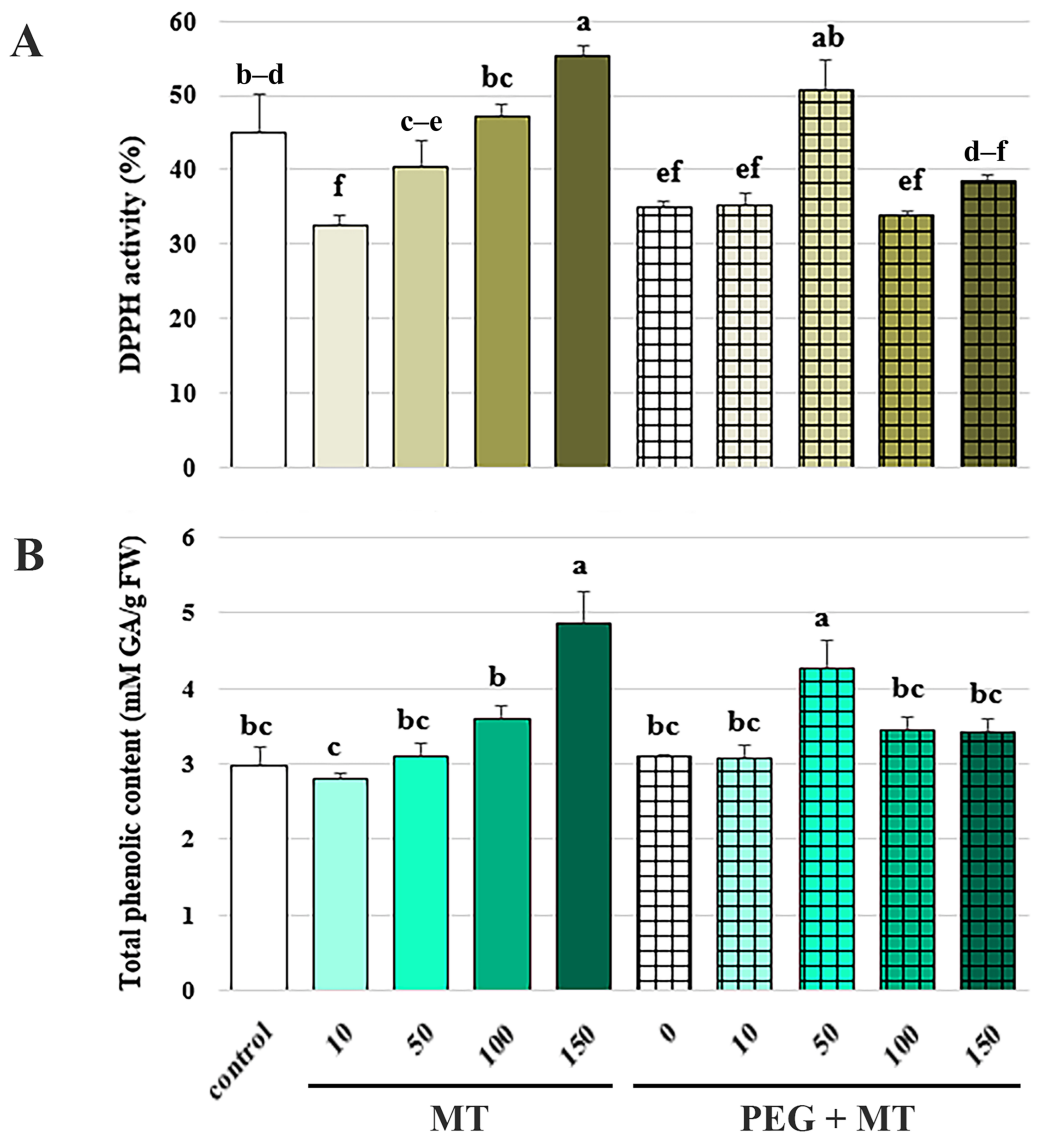
Effects of exogenously applied melatonin under optimal and water-deficit (3% PEG) conditions in vitro on the degree of DPPH radical reduction (**A**) and TPC (**B**) in *I. walleriana* shoots. Data represent mean ± SE (*n* = 21). Different letters indicate significant differences between treatments (LSD test, *p* ≤ 0.05).

As shown in Figure 7A, the activity of the DPPH radical scavenger varied with the applied MT concentration. In *I. walleriana* shoots grown on MS medium with 10 µM MT, DPPH activity significantly decreased compared to the control plants. In contrast, plants grown on medium containing 150 µM MT showed significantly higher DPPH activity. Water deficit, alone or combined with 10 or 100 µM MT, significantly reduced DPPH activity compared to the control, while shoots grown under water-deficit stress on medium with 50 µM MT resulted in a certain, but not significant, increase in DPPH activity.

The total polyphenol content (TPC) also changed depending on the treatment (Figure 7B). The addition of 10, 50 and 100 µM MT did not significantly change TPC in comparison to the control *I. walleriana* shoots. The only significant increase in TPC was observed in shoots grown on culture medium containing 150 µM MT. Water deficit, alone or in combination with MT, also did not significantly change TPC in *I. walleriana* shoots. The exception was shoots cultivated on 50 µM MT, in which, during water stress, a significant increase in TPC was detected. Thus, under optimal conditions, only 150 µM MT increased the antioxidant capacity and polyphenol accumulation in *I. walleriana* shoots. Under water-deficit stress, 50 µM MT stimulated the antioxidant defence mechanism as well as polyphenol accumulation, while lower or higher MT concentrations were less effective.

### 2.6. Activity of Antioxidant Enzymes

**Figure 8:**
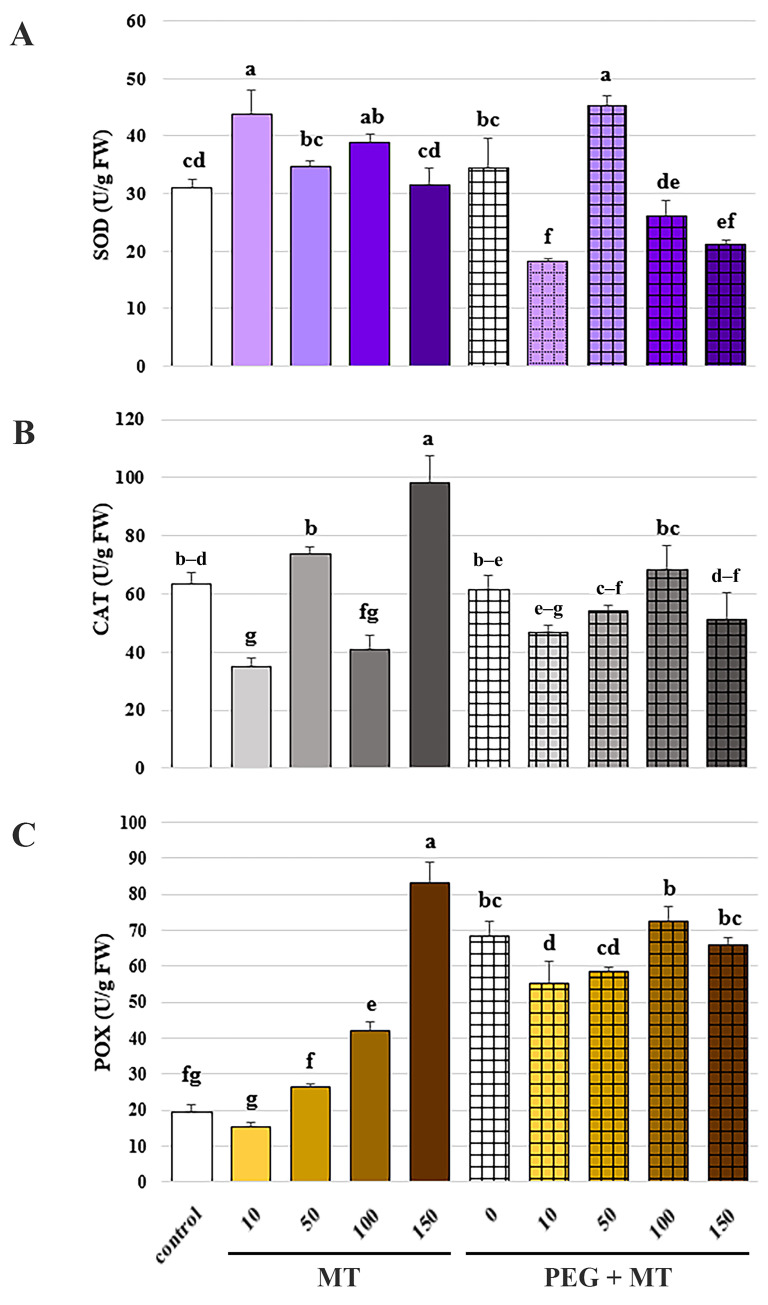
Effects of exogenously applied melatonin under optimal and water-deficit (3% PEG) conditions in vitro on SOD (**A**), CAT (**B**) and POX (**C**) activity in *I. walleriana* shoots. Data represent mean ± SE (*n* = 21). Different letters indicate significant differences between treatments (LSD test, *p* ≤ 0.05).

The exogenous application of MT significantly altered the activity of antioxidant enzymes in *I. walleriana* shoots (Figure 8). Our results showed that applications of 10 and 100 µM MT significantly increased SOD activity (Figure 8A), but decreased CAT activity (Figure 8B), compared to the control. A concentration of 100 µM MT also stimulated an increase in POX activity in *I. walleriana* shoots (Figure 8C). However, the maximum activities of CAT and POX were observed in *I. walleriana* shoots grown on medium with 150 µM MT, suggesting that high MT concentrations enhance the antioxidant response in plants. The highest SOD activity was recorded in plants grown on culture medium containing 10 µM MT.

During water deficit, exogenous applications of 10 and 150 µM MT significantly reduced SOD activity (Figure 8A). The only increase in SOD activity during water deficit occurred in *I. walleriana* shoots grown on medium containing 50 µM MT, which was also the highest SOD activity recorded. Water deficit combined with 10 and 50 µM MT significantly decreased CAT activity (Figure 8B). Our results indicate that POX activity significantly increased during water deficit alone but also in water-deficit treatment combined with all applied MT concentrations compared to control shoots (Figure 8C). In addition, water deficit combined with 100 µM MT further increased POX activity (72.43 U/g FW), representing the maximum activity of this enzyme during water deficit and a significant increase compared to the control.

## 3. Discussion

### 3.1. Melatonin and I. walleriana Morphological Traits Under Water Deficit

Drought, as one of the most important environmental factors, affects the vegetative growth and productivity of plants, whether grown in the open field, under ex vitro conditions [46], or in vitro [47]. *I. walleriana* is sensitive to water deficiency in the medium, and treatment by PEG leads to a significant reduction in shoot height, the number of leaves per plant, and shoot biomass [48]. During recent years, different strategies were explored to improve drought tolerance in this ornamental plant species [49]. This work continues the investigation into the potential protective effect of MT on *I. walleriana* shoots grown under water deficit in vitro.

Melatonin has a dual role in plants: stimulating growth and development while also providing protection from abiotic stresses [41,50,51,52,53]. Results reported by Posmyk and Janas [54], Arnao and Hernández-Ruiz [55], Yang et al. [56], and Amiri et al. [51] indicated that low concentrations of MT enhance elongation and cell division, accelerated shoot growth and increased biomass accumulation. Data from the recent literature observed that MT could have positive effect on plants during drought stress. For example, the exogenous application of MT alleviates drought stress in *Z. mays* by maintaining plant growth, improving photosynthetic characteristics and antioxidant enzyme activities. The most effective treatment concentration was the foliar application of 100 µM MT and substrate-wetting with 50 µM MT [34]. Treatment of *Lycopersicon esculentum* seedlings with 100 µM MT improved tolerance to drought stress, with increased plant growth and altered root architecture [39]. Li et al. [57] showed that a concentration of 100 μM MT, used to coat *Triticum aestivum* seeds, was the most effective in protecting plants from drought, as it significantly increased fresh weight, plant height and root length. In our study, treatment with MT affected the growth parameters of *I. walleriana* in a concentration-dependent manner under both optimal and water-deficit conditions. The application of MT at a dose of 10 µM positively affected shoot height, the number of leaves formed and fresh shoot weight of *I. walleriana* under optimal conditions, while only shoots grown with the same MT concentration in combination with PEG formed the same number of leaves as the control. Higher concentrations of MT inhibited the growth and development of *I. walleriana* under both optimal and water-deficit conditions. This improvement in plant growth can be attributed to increased synthesis and the increased accumulation of auxins and cytokinins, which stimulate cell division and elongation, as well as the regulation of these hormonal signalling pathways mediated by MT [58]. However, our results showed that concentrations of 50 μM MT and above reduced shoot height, the number of newly formed shoots, and the fresh weight of shoots and roots under both optimal and water-deficit conditions (Figure 1 and Figure 2). This is consistent with the findings that excessive MT can disrupt hormonal balance, leading to growth suppression, which could be due to metabolic imbalances or the toxicity of MT at elevated concentrations [35,59].

In addition, 0.1 µM MT has a stimulatory effect on root elongation in 2-day-old *Brassica juncea* seedlings, but higher concentrations, such as 100 µM MT, inhibit root elongation in young seedlings, while 4-day-old seedlings are not susceptible to either the stimulatory or inhibitory effects of MT [60]. David et al. [40] observed a dramatic increase in the height of *M. citrifolia* plants grown under optimal conditions when treated with 50 μM MT. The same authors also observed a progressive increase in the height of *M. citrifolia* with increasing MT concentrations from 0, 50 and 100 μM when treated with 40% PEG [40]. The results of our study showed that under optimal growth conditions, increasing MT concentration slows root development (Figure 1 and Figure 2). Even on medium with 10 μM MT, the fresh root weight of *I. walleriana* was significantly lower than in the control and at 50 μM, the rootlet was barely visible, while no roots were formed on shoots grown at 100 μM. In shoots of *I. walleriana* grown only under water-deficit conditions, the root was significantly smaller than in control plants, as indicated by a significant decrease in root weight. In shoots treated with 10 μM MT and PEG, the morphology and fresh root weight did not differ significantly from those formed under water-deficit conditions (Figure 1 and Figure 2). In contrast, on the PEG and MT 50 μM, the root was barely visible, and higher concentrations of MT did not induce the development of roots at all. These results indicate that MT does not have a protective function in *I. walleriana*; rather, at higher concentrations, it causes stress in *I. walleriana* in shoots grown under both optimal and water-deficit conditions. Whether treatments of *I. walleriana* with concentrations lower than 10 µM MT would have a stress-alleviating effect remains to be investigated. Amiri et al. [51] also found that the treatment of *Trigonella foenum-graecum* with MT (100, 300 and 500 μM) under water-deficit conditions inhibited root growth. Contrary to the above, foliar treatment of *Ranunculus asiaticus* with 200 µM MT under water-deficit conditions induced bud emergence 21 days earlier than in control plants and caused a series of physiological changes, indicating the effective role of high concentrations of MT in improving the adaptability of *R. asiaticus* to drought stress [41]. Ge et al. found that 200 μmol/L MT was the optimal concentration for alleviating drought stress in *Camellia hainanica* seedlings, making it suitable for practical use in *C. hainanica* production [61]. Taken together, these findings confirm the dual role of MT as a growth-promoter and potential inhibitor, depending on the applied concentration. Thus, when determining the beneficial effects of MT, it is necessary to optimise the concentration for each plant species tested before MT has any practical application in horticulture, forestry or agriculture.

### 3.2. Melatonin and Photosynthetic Pigments Under Water Deficit

The key role of MT in regulating stress is the protection of photosynthetic mechanisms and stimulation of tissue proliferation [62], which places MT in a superior position compared to ascorbate and tocopherol in terms of antioxidant properties. It is well known that MT can improve photosynthetic activity by increasing chlorophyll content or photosystem II quantum efficiency (Fv/Fm), under either optimal or stress conditions [63,64,65]. In drought conditions, the treatment of *Glycine max* with MT at low to moderate concentrations prevented pigment degradation and improved stress tolerance [36]. Under optimal conditions, 50 and 100 µM MT increased *Chl a* and *Chl b* content in *M. citrifolia* [40]. On the other hand, when this plant is grown at 20% PEG, both concentrations lead to an increase in *Chl a* content, while under more intense water deficit (40% PEG), 50 µM MT increased the content of both *Chl a* and *Chl b* [40]. In our experiment, 10 µM MT caused a decrease in *Chl a* and *Chl b* content under optimal conditions, and a sharp decrease in *Chl a*, *Chl b* and total chlorophyll during water-deficit conditions. Additionally, the higher MT concentrations further decreased chlorophyll content (Figure 3 and Figure 4), which is in contrast with the generally protective effects reported in the literature [40,59]. These findings confirm the ambiguous role of MT in modulating plant responses under both optimal and stress conditions.

The *Chl a*/*Chl b* ratio in *I. walleriana* was relatively stable under MT treatments in optimal growth conditions, as well as with the application of 10 and 50 µM MT under stress conditions. The higher MT concentrations (100 and 150 µM) during water deficit significantly decreased the *Chl a*/*Chl b* ratio (Figure 3C), indicating the degradation of chlorophyll. This imbalance is often associated with damage to photosystem II or reduced stability of the light-harvesting complex [66]. During drought, the decrease in chlorophyll content is associated with the oxidative destruction of chloroplast membranes or increased pigment degradation [67]. The activity of the enzymes chlorophyllase (CHLASE), pheophytinase (PPH), chl-degrading peroxidase (Chl-PRX), and pheophorbide–oxygenase (PAO), which are involved in chlorophyll degradation, increases during drought [68]. Although it is confirmed that MT downregulates these enzymes under stress conditions and upregulates chlorophyll metabolites [66,68], our results suggest that in *I. walleriana*, high MT concentrations under severe osmotic stress may unexpectedly interfere with pigment biosynthesis pathways and decrease chlorophyll content. Overall, the results indicate that the effect of MT application depends on the concentration used, where the highest concentration of MT negatively affects the content of all pigments in plants exposed to stress.

The trend in carotenoid content changes was like that of chlorophyll content in *I. walleriana*, with the most significant decrease observed in the treatment with 100 µM MT during water deficit (Figure 4B). As carotenoids play a vital role in photoprotection and scavenging ROS, they effectively quench singlet oxygen and interrupt chain reactions of free radicals [69], their reduction at high MT concentrations combined with water deficit may indicate the excessive stimulation of metabolic stress responses or impaired antioxidant signalling. According to Wang et al., the inhibitory effect of exogenously applied MT on pigment metabolism, especially carotenoid synthesis during stress, may result from the interaction of MT with endogenous ABA [70].

### 3.3. Melatonin and I. walleriana Cell Oxidative Biomarkers Under Water Deficit

A defence system composed of non-enzymatic and enzymatic antioxidants plays an essential role in protecting chloroplasts and mitochondria from oxidative stress caused by accumulated ROS under abiotic or biotic stress [68,71]. The production of osmolytes (proteins, carbohydrates, proline and total free amino acids) that have a protective role plays a crucial role in reducing the harmful effects of water deficiency [72]. Proline, which serves as a stress indicator, contributes to osmoregulation (effectively protecting the stability of the cell membrane) by scavenging ROS, and acting as a cellular redox buffer. It also serves as a carbon and nitrogen store and plays a vital role in signal transduction and the regulation of mitochondrial function [50]. Cao et al. found that drought significantly increased proline content in *G. max*, and that the exogenous application of MT during stress further promoted this increase [73]. In this way, proline helps plants to maintain normal cellular osmotic potential, reduce water loss, and support the normal functioning of all cellular organelles [73]. In this study, shoots accumulated proline under water-deficit conditions, and with an increase in MT concentration, the proline content was also increased under both optimal and drought conditions (Figure 5A). Similar results were also recently reported, confirming that the application of 100 μM MT significantly increased proline content and antioxidant enzyme activity in *Oryza sativa*, *Citrus latifolia* and *C. aurantifolia* under drought stress [74,75]. In addition to proline, the graded MT concentrations also elevated the total amino acid content in *I. walleriana*, especially under water deficit (Figure 5B), suggesting that both factors together stimulate amino acid synthesis. *I. walleriana* shoots grown on 10–100 µM MT showed a significantly lower proportion of proline in the total amino acid content compared to the control, due to the increase in amino acid production. Similarly, drought stress increased the synthesis and accumulation of proline and several amino acids in *Z. mays* roots, while MT treatment further increased total amino acid levels in both roots and leaves of drought-stressed plants [76]. These results showed that drought-stressed plants accumulate more amino acids, particularly in roots compared to leaves, suggesting a strategic adaptation of *Z. mays* to environmental challenges, as amino acids act as precursors for signalling molecules that influence other substances regulating the stress response. Thus, the accumulation of proline and other amino acids increases the survival ability of plants during stress by detoxifying them, acting as antioxidants, and stabilising macromolecules through scavenging free radicals, buffering cellular redox potential, and maintaining the integrity of plasma membranes [41].

Although lower concentrations of ROS in the cell are not harmful, but instead participate in cell as signalling agents [38,77], their increase can damage the structural integrity of plant cell membranes. This occurs due to the interaction of ROS with phospholipids and fatty acids, which accelerates membrane lipid peroxidation and produces toxins such as MDA [78]. Hydrogen peroxide is a reactive oxygen species produced by plant cells, whose levels increase under abiotic stress conditions. Therefore, clear indicators of oxidative stress in plants exposed to water deficit are increased levels of H_2_O_2_ and MDA [79]. Under optimal growth conditions, the application of 50 and 150 μM MT concentration in *I. walleriana* was stressful for the plants and led to H_2_O_2_ accumulation. Namely, both water deficit alone and the application of 50 and 150 µM MT during water deficit led to an increase in H_2_O_2_ content in *I. walleriana* shoots compared to control plants (Figure 6A), indicating a higher risk of oxidative damage. Similarly, in *T. aestivum* seed treatment with MT, low concentrations (25 and 50 μM) inhibited the formation of H_2_O_2_; however, when MT increased to 100 μM, the H_2_O_2_ content also increased significantly [57]. Thus, MT not only participates in the elimination of H_2_O_2_ but also can induce H_2_O_2_ production when its concentration exceeds a certain level [57,80]. In addition, the reduction in MDA content in *I. walleriana* shoots was determined only in shoots grown on medium with 10 µM MT under optimal growing conditions, while all other treatments did not affect MDA content (Figure 6B). The decrease in the degree of lipid peroxidation observed in *I. walleriana* was significantly smaller than the changes reported by Neamah and Jdayea in *Hyoscyamus pusillus callus* [43]. The treatment of *H. pusillus* callus with 4% PEG resulted in a significant increase in H_2_O_2_ and MDA content, leading to oxidative stress in the tissue. Also, treating callus with a high (1.5 mg/L) MT during water deficit resulted in a 97.23% decrease in H_2_O_2_ content and a 2.25-fold decrease in MDA content compared with their concentrations in callus grown under water stress alone [43]. A similar effect was observed in *Moringa oleifera* foliar treated with 100 mM MT under both optimal and drought conditions [64]. In *C. latifolia* and *C. aurantifolia* plants under drought stress, MT treatment significantly reduced MDA levels and membrane leakage, while MT-treated plants also exhibited a decrease in H_2_O_2_ concentration [75].

The degree of DPPH radical reduction is used to determine the antioxidant capacity of plants. In this work, DPPH radical scavenging activity and polyphenol accumulation in *I. walleriana* shoots were directly dependent on the applied concentration of MT, both under optimal conditions and PEG-induced water deficit. *I. walleriana* shoots grown under optimal conditions and treated with a low concentration of MT (10 µM) showed reduced levels of oxidative stress markers compared to control plants, while the application of 150 µM MT significantly increased DPPH activity and TPC (Figure 7). These findings are consistent with previous reports [81,82,83], which indicated that high doses of MT could increase the content of non-enzymatic antioxidants, such as phenolic compounds, flavonoids and ascorbate, in *Cucumis sativus* and *Ocimum basilicum*. The results obtained in *I. walleriana* shoots grown with 150 µM MT confirm the ability of MT to activate the phenylpropanoid pathway, thereby enhancing the biosynthesis of secondary metabolites with antioxidant properties. In *I. walleriana* grown on PEG medium, DPPH activity was suppressed, reflecting oxidative stress induced by osmotic imbalance. Under water deficit conditions, a certain increase in the ability of *I. walleriana* to reduce DPFH was observed with the 50 µM MT treatment (Figure 7A). In this treatment, a significant increment in proline and total phenolic content was also detected, indicating that the enhanced antioxidative capacity of both enzymatic and non-enzymatic antioxidants contributed to plant defence against stress due to accumulated H_2_O_2_ (Figure 5, Figure 6 and Figure 7). Despite this, the almost completely underdeveloped roots and reduced pigment concentration contributed to the poor morphological appearance of these plants (Figure 1). Similar results were reported by Li et al., who found that exogenous MT at moderate levels improved antioxidant defence in drought-stressed *S. lycopersicum* and *Z. mays* by increasing the biosynthesis of polyphenols [84]. Other concentrations of MT in the medium during water deficit failed to significantly improve DPPH activity in *I. walleriana*, indicating a narrow optimal MT concentration range for enhancing antioxidant activity. This phenomenon was also observed by Tan et al., who emphasised that both insufficient and excessive levels of MT can disrupt cellular redox balance or lead to feedback inhibition of biosynthetic enzymes [85]. Under optimal conditions, the TPC of *I. walleriana* increased with higher concentrations of applied MT. Under water-deficit conditions, only treatment with 50 µM MT resulted in a significant increase in TPC in the shoots of *I. walleriana* (Figure 7B). This result supports the hypothesis that moderate application of MT increases the accumulation of phenolic compounds under adverse conditions, as has also been observed in *Vitis vinifera*, *Cucumis sativus* and *Triticum aestivum* under drought and salt stress [86,87].

### 3.4. Melatonin and I. walleriana Antioxidant Enzyme Activity Under Water Deficit

For plant survival under drought conditions, it is essential to maintain a balance between ROS production and their removal by enzymes or other components of the antioxidant defence system to achieve stress tolerance [88]. The excessive accumulation of ROS leads to oxidative stress, which changes the activity of antioxidant enzymes in plants [36]. The effect of MT on enhancing plant tolerance to stress lies in its ability to improve the balance between ROS production and removal through changes at the physiological, biochemical, and molecular levels [8,32]. In *I. walleriana* under optimal growth conditions, the application of 10 µM MT led to the most pronounced increase in SOD activity (Figure 8A). However, under water-deficit conditions, increased SOD activity was present only at 50 µM MT. This treatment also increased the content of proline, total amino acids, H_2_O_2_, and TPC, which together provided the highest degree of protection for *I. walleriana* against stress. SOD, as the first line of plant defence against stress, effectively maintains H_2_O_2_ homeostasis and thus protects cell membranes from lipid peroxidation. SOD plays a major role in scavenging ROS and converting O^−2^ to O_2_ and H_2_O_2_, after which POX and CAT decompose H_2_O_2_ to H_2_O and O_2_ [89,90]. In *Salvia nemorosa* and *Salvia reuterana*, after treatment with MT under drought conditions, higher SOD activity in *S. nemorosa* was correlated with better drought tolerance [37]. The results of Zhang et al. showed that treating *Oryza sativa XZX45* seeds exposed to drought with 100 μM MT effectively improved germination potential, rate, and index, increased the activities of SOD, POX, and CAT, and reduced the content of MDA, thereby maintaining normal cellular metabolism and alleviating drought stress [91]. The research of Cao et al. indicated that drought (45% relative soil water content) increased the activity of SOD, POD, and CAT in *G. max* leaves, while treatment with exogenous MT combined with drought further increased the activity of these enzymes [36]. Therefore, the protective effect of MT against drought-induced stress can be attributed to its antioxidant capacity and free radical scavenging. In *Morinda citrifolia* grown in vitro, maximum CAT activity was measured in plants cultivated on MS medium with 40% PEG and 50 μM MT, while the highest GPX and SOD activities were recorded in samples treated with 20% PEG and 50 μM MT [40]. Similarly, Imran et al. reported that *G. max* plants exposed to drought stress showed increased H_2_O_2_ accumulation and decreased CAT, PPO, POX, APX, and SOD activities [63]. However, the treatment of *G. max* with 100 μM MT by foliar or root irrigation under stress conditions resulted in a significant increase in antioxidant enzyme activities and the inhibition of H_2_O_2_ accumulation. The potential of exogenous MT administration to alleviate stress depends on its concentration and the method of application. In *I. walleriana* grown on medium with 150 µM MT under optimal conditions, the maximum values of H_2_O_2_, TPC, and DPPH, and high levels of proline were confirmed, and an increase in CAT and POX activities was determined (Figure 1, Figure 2, Figure 5, Figure 6, Figure 7 and Figure 8). The results indicate a strong negative impact of the highest MT concentration on the physiological processes of *I. walleriana* and high sensitivity to treatment. *I. walleriana* shoots treated with 150 µM MT under water-deficit conditions showed low pigment contents, but increased amino acids and H_2_O_2_ content, and an increase in POX activity (Figure 3, Figure 4, Figure 5, Figure 6 and Figure 8). Although, under abiotic stress, the activity of SOD, CAT, and POX is frequently increased simultaneously, some plants do not need the initiation of all antioxidant enzymes for drought tolerance. While these enzymes work together, their responses can differ; sometimes, CAT activity increases while POX activity remains unchanged or declines, or vice versa [92,93]. For instance, in the drought-tolerant *Amaranthus tricolor* variety, only SOD, CAT and AsA-GSH cycle enzymes play a vital role in ROS detoxification. However, proline and POX accumulation were higher in the drought-sensitive *A. tricolor* genotype [92]. The increase in proline content and POX activity in *R. asiaticus* after the exogenous application of MT under stress conditions indicates these parameters serve as markers of drought tolerance [50]. These findings further confirm previous reports in *C. arabica* [35], *G. max* [94], *S. lycopersicum* [39], *M. citrifolia* [40], *H. pusillus* [43], *Prunus persica* [95] and *T. aestivum* [56]. In conclusion, this study clarifies the changes in *I. walleriana* shoots induced by MT treatment under optimal conditions and water-deficit conditions, focusing on antioxidant capacity. The results provide an experimental basis for further research on this sensitive species using MT at concentrations below 10 µM.

## 4. Materials and Methods

### 4.1. Plant Materials and Experimental Design

The starting material for the experimental work was an in vitro culture of *I. walleriana* (“Xtreme Scarlet”, Syngenta Flowers, Enkhuizen, The Netherlands), grown over thirty days on Murashige and Skoog medium (MS) [96]. The solid nutrient medium contained mineral salts, vitamins, 30 g/L sucrose, 100 mg/L myo-inositol (Sigma Aldrich, St. Louis, MO, USA) and 7 g/L agar (Institute of Virology, Vaccines, and Serums, “Torlak”, Belgrade, Serbia). After adjusting the pH of the medium to 5.6 (Edge pH manual, Hanna Instruments, Belgrade, Serbia), it was autoclaved at 114 °C for 20 min. The *I. walleriana* cultures were grown in the growth chamber under long-day conditions (16/8 h light/darkness) at an irradiance of 47 μmol/m^2^ s^1^ and a temperature of 25 ± 2 °C. The starting material for the experimental setup consisted of apical shoot segments ≈ 2.5 cm high. These explants were isolated from plants grown on MS medium, with regular subculture every twenty-eight days (Table 1). The *I. walleriana* shoots grown on MT-free medium served as the control group. One group of plants was grown on MS medium supplemented with 0, 10, 50, 100 or 150 µM MT, while the other group was grown under the same MT concentrations in combination with water-deficit conditions induced by the addition of 3% polyethylene glycol (PEG_8000_, Sigma Aldrich, St. Louis, MO, USA). A stock solution of MT (Sigma Aldrich, St. Louis, MO, USA) was prepared by dissolving it in 96% ethanol (Zorka Pharma-Hemija DOO, Šabac, Serbia), then further diluted with sterile deionised water under aseptic conditions. These working solutions were added to the cooled MS medium after sterilisation. Since 96% ethanol was used to dissolve MT, an equivalent amount of ethanol was added to the distilled water, and then to the MS medium used for growing control plants. After twenty-eight days of cultivation, morphological parameters were measured and shoot samples were collected, frozen in liquid nitrogen and stored at −80 °C for further analyses. This experiment was repeated three times. We selected 3% PEG for water-deficit induction based on the results regarding the sensitivity of *I.* *walleriana* to drought stress in vitro, published by Antonić et al. [47].

### 4.2. Growth Parameters

Twenty-eight days after the experimental setup, plants were carefully removed from the glass jars. The remaining nutrient medium was discarded and the roots were washed to remove any residual medium. The washing water was collected using filter paper, and the shoots were separated from the roots. The shoot length (cm) was measured with a ruler while the fresh weight (FW) of shoots and roots (g) was determined using a digital scale. The number of developed leaves and newly formed shoots was also recorded.

### 4.3. Determination of Photosynthetic Pigments Content

The method of Lichtenthaler (1987) was used to determine the content of chlorophyll a (*Chl a*), chlorophyll b (*Chl b*), total chlorophyll and carotenoid in the *I. walleriana* leaves [97]. The leaves were immersed in 96% ethanol and incubated in a water bath at 70 °C (Univeba JP Selecta, Barcelona, Spain). The absorbance of photosynthetic pigments was measured at 470, 648 and 664 nm using a UV–visible spectrophotometer (Shimadzu UV-1900i, Kyoto, Japan). The results were expressed in mg/g FW of the sample.

### 4.4. Determination of Oxidative Stress Indicators

#### 4.4.1. Quantification of Proline and Free Amino Acids

To determine the content of free proline, the ninhydrin reaction was used according to a modified method described by Friedman [98]. As a result of the reaction of proline contained in the sample and the ninhydrin reagent (Sigma Aldrich, St. Louis, MO, USA), a yellow reaction product was formed. The ELISA Micro Plate Reader (LKB 5060-006, Winooski, VT, USA) was used to measure the content of free proline and amino acids in the samples at 350 and 570 nm, respectively. The calculations were performed according to the method described by Carlo and Gibbon [99], and the results were expressed in µM/g FW.

#### 4.4.2. Hydrogen Peroxide Content and Lipid Peroxidation

The amount of H_2_O_2_ was measured according to the method developed by Velikova et al. [100], in which H_2_O_2_, from plant tissue homogenised in cooled trichloroacetic acid (TCA, Sigma Aldrich, St. Louis, MO, USA), was reacted with potassium iodide. The optical absorbance of the supernatant was measured at 390 nm using the ELISA Micro Plate Reader (LKB 5060-006, Winooski, VT, USA), and expressed as µg/g FW.

The product of lipid peroxidation, MDA, was determined spectrophotometrically according to the method of Heath and Packer [101]. The quantification of MDA, which is formed during the reaction of lipid peroxide with thiobarbituric acid (TBA, Sigma Aldrich, St. Louis, MO, USA), is carried out at 532 nm and expressed as nM/g FW. Both methods have previously been described in detail [102].

#### 4.4.3. DPPH Radical Scavenging Capacity Assay and Total Polyphenol Content

The ability of *I. walleriana* plants to scavenge free radicals was measured using the method of Brand-Williams et al. [103]. A purple methanol solution of the free radical 1,1-diphenyl-2-picrylhydrazyl (DPPH, Sigma Aldrich, St. Louis, MO, USA) was used. When antioxidants from methanol extracts of the plants reacted with DPPH after incubation in the dark, the DPPH radical was reduced to DPPH-H, resulting in a yellow colour. The degree of DPPH radical reduction (%) was determined by measuring the absorbance at 520 nm (Shimadzu UV-1900i, Kyoto, Japan). The detailed protocols were previously described by Subotić et al. [102].

The determination of total phenolic compounds (TPC) was carried out according to the method developed by Singleton et al. [104]. Total polyphenols from methanol extracts reacted with the Folin–Ciocalteu reagent (FC, Sigma Aldrich, St. Louis, MO, USA) to form a blue-coloured complex, and quantification was performed by measuring the absorbance of the reaction mixture at 765 nm using gallic acid as the phenol standard (Shimadzu UV-1900i, Kyoto, Japan). As with the previous method, Subotić et al. [102] provided a detailed description of the determination of TPCs.

### 4.5. Protein Extraction and Determination of Antioxidant Enzyme Activities

For the extraction of total soluble proteins, a sample of fresh shoot tissue (1 g) was ground to a fine powder in liquid nitrogen with 1 mL of cold protein extraction buffer (PEB), using the procedure described by Milošević et al. [105]. The supernatant was aliquoted, stored at −80 °C and used to analyse the activities of three antioxidant enzymes: SOD (SOD; EC 1.15.1.1), CAT (CAT; EC 1.11.1.6) and POX (POX; EC 1.11.1.7).

Total SOD activity was measured according to the protocol of Beyer and Fridovich [106], with modifications as described by Antonić et al. [48]. The reaction is based on the photoreduction of nitro-blue tetrazolium (NBT) by the plant extract to formazan, which is blue, in the presence of EDTA, methionine, riboflavin, and phosphate buffer. Absorbance was measured at 540 nm using an ELISA microplate reader (Agilent BioTek Synergy H1 Multimode Reader, Santa Clara, CA, USA). One SOD unit was defined as the amount of enzyme required to inhibit 50% of NBT photoreduction compared to the reaction mixture without sample.

Total CAT activity was measured by spectrophotometric monitoring of the kinetics of H_2_O_2_ consumption by the sample extract, following the method described by Aebi [107]. The reaction was monitored at 240 nm (Agilent 8453 spectrophotometer, Santa Clara, CA, USA) by measuring the change in absorbance (∆A) over time (∆t) during the linear part of the curve.

Total POX activity was determined by Vuleta et al. [108]. The reaction was based on the transfer of electrons from pyrogallol to H_2_O_2_, catalysed by peroxidase. In this reaction, pyrogallol is oxidised to form a yellowish-brown compound, purpurogallin, which has an absorption maximum of 430 nm (UV–visible spectrophotometer Shimadzu UV-160, Kyoto, Japan). One unit (1 U) of POX activity is defined as the amount of enzyme that produces 1 µmol of product per minute.

The SOD, CAT and POX activities are expressed per gram of fresh weight (U/g FW).

### 4.6. Statistical Analysis

The analysed parameters were evaluated for seven plants per treatment. All experiments were repeated three times (*n* = 21), and the results are presented as the mean ± standard error. To assess the statistical differences between experimental treatments, a standard analysis of variance (ANOVA) was performed using STATISTICA software version 8. The mean differences were compared using the least significant difference (LSD) test, with statistical significance set at *p* < 0.05. Microsoft Office Excel (2010) was used for graphical presentation of the results.

## 5. Conclusions

Numerous studies indicate that MT, a natural, non-toxic biostimulant, can be used in horticulture and agriculture without negative effects on the environment. Exogenous application of MT could increase plant productivity and quality but also helps plants to survive stressful environmental conditions. This report provides insight into the effects of MT on the antioxidant capacity of *I. walleriana* shoots grown in vitro under water-deficit conditions induced by PEG application. Our results confirm that the studied species, in addition to being sensitive to water deficit, also shows high sensitivity to the applied MT concentrations. *I. walleriana* is one of the most widely cultivated ornamental species worldwide, and future studies should be conducted using MT at concentrations below 10 µM for in vitro culture or 10 µM for foliar treatment. As MT is a central molecule in the hormonal system and orchestrates other phytohormones within the regulatory defence network during plant tolerance to water-deficit stress, our future research will focus on analysing the content of endogenous phytohormones, especially MT, in *I. walleriana* shoots grown on media with varying, even lower concentrations of MT. Likewise, investigating the expression of genes responsible for antioxidant enzymes would also be of great importance.

## Figures and Tables

**Table 1:** Experimental setup of the MT and PEG treatments.

Treatments									
control	µM MT				µM MT + 3% PEG				
	10	50	100	150	0	10	50	100	150

## Data Availability

The original contributions presented in this study are included in the article. Further inquiries can be directed to the corresponding author.

## Abbreviations

The following abbreviations are used in this manuscript:

MT	Melatonin, (N-acetyl-5-methoxytryptamine)
PEG	Polyethylene glycol
ROS	Reactive oxygen species
RNS	Reactive nitrogen species
H_2_O_2_	Hydrogen peroxide
MDA	Malondialdehyde
*Chl a*	Chlorophyll a
*Chl b*	Chlorophyll b
DPPH	1,1-diphenyl-2-picrylhydrazyl
TPC	Total phenolic compounds
SOD	Superoxide dismutase
CAT	Catalase
POX	Peroxidase
IAA	indole-3-acetic acid
TCA	Trichloroacetic acid
TBA	Thiobarbituric acid
FC	Folin–Ciocalteu reagent
NBT	Nitro-blue tetrazolium
